# Supplementary material for: Multi‐dataset identification of innovative feature genes and molecular mechanisms in keratoconus
Source: J Cell Mol Med. 2024 Sep 19;28(18):e70079. doi: 10.1111/jcmm.70079 (PMC11412914; doi:10.1111/jcmm.70079)
Supplement: Supplementary file 2 — Material S2. [file JCMM-28-e70079-s002.zip › 7.GSEA analysis/2.ATOH7/KEGG_ACUTE_MYELOID_LEUKEMIA.html]

Details for gene set KEGG\_ACUTE\_MYELOID\_LEUKEMIA[GSEA]

|  || Dataset | ATOH7.group.cls#h\_versus\_l |
| Phenotype | group.cls#h\_versus\_l |
| Upregulated in class | h |
| GeneSet | KEGG\_ACUTE\_MYELOID\_LEUKEMIA |
| Enrichment Score (ES) | 0.36219707 |
| Normalized Enrichment Score (NES) | 1.5181866 |
| Nominal p-value | 0.03649635 |
| FDR q-value | 0.24937554 |
| FWER p-Value | 0.751 |
Table: GSEA Results Summary

  

Fig 1: Enrichment plot: KEGG\_ACUTE\_MYELOID\_LEUKEMIA      
 Profile of the Running ES Score & Positions of GeneSet Members on the Rank Ordered List

  

| SYMBOL | TITLE | RANK IN GENE LIST | RANK METRIC SCORE | RUNNING ES | CORE ENRICHMENT || 1 | AKT3 | na | 9 | 1.043 | 0.1473 | Yes |
| 2 | LEF1 | na | 735 | 0.492 | 0.1960 | Yes |
| 3 | CCNA1 | na | 2422 | 0.314 | 0.1918 | Yes |
| 4 | ZBTB16 | na | 2887 | 0.286 | 0.2190 | Yes |
| 5 | TCF7 | na | 3059 | 0.277 | 0.2532 | Yes |
| 6 | STAT5A | na | 3484 | 0.257 | 0.2773 | Yes |
| 7 | MAP2K1 | na | 4240 | 0.228 | 0.2877 | Yes |
| 8 | PIM2 | na | 5969 | 0.172 | 0.2621 | Yes |
| 9 | RUNX1 | na | 6118 | 0.168 | 0.2816 | Yes |
| 10 | PIK3R3 | na | 6166 | 0.166 | 0.3037 | Yes |
| 11 | RELA | na | 6248 | 0.164 | 0.3245 | Yes |
| 12 | TCF7L1 | na | 6644 | 0.153 | 0.3348 | Yes |
| 13 | EIF4EBP1 | na | 6778 | 0.149 | 0.3520 | Yes |
| 14 | RUNX1T1 | na | 7399 | 0.133 | 0.3529 | Yes |
| 15 | NRAS | na | 7693 | 0.126 | 0.3622 | Yes |
| 16 | NFKB1 | na | 8680 | 0.105 | 0.3486 | No |
| 17 | ARAF | na | 10150 | 0.079 | 0.3174 | No |
| 18 | GRB2 | na | 10441 | 0.075 | 0.3196 | No |
| 19 | STAT3 | na | 11584 | 0.058 | 0.2948 | No |
| 20 | KIT | na | 11952 | 0.053 | 0.2917 | No |
| 21 | RARA | na | 13254 | 0.038 | 0.2595 | No |
| 22 | PIM1 | na | 13639 | 0.034 | 0.2533 | No |
| 23 | MAPK1 | na | 14536 | 0.025 | 0.2310 | No |
| 24 | HRAS | na | 14559 | 0.025 | 0.2339 | No |
| 25 | RAF1 | na | 15219 | 0.019 | 0.2177 | No |
| 26 | PML | na | 15223 | 0.019 | 0.2203 | No |
| 27 | IKBKG | na | 15619 | 0.016 | 0.2111 | No |
| 28 | JUP | na | 15727 | 0.015 | 0.2102 | No |
| 29 | AKT1 | na | 15787 | 0.014 | 0.2105 | No |
| 30 | PIK3CD | na | 16202 | 0.011 | 0.2001 | No |
| 31 | MAPK3 | na | 16821 | 0.006 | 0.1832 | No |
| 32 | PPARD | na | 17078 | 0.004 | 0.1763 | No |
| 33 | BAD | na | 17218 | 0.002 | 0.1727 | No |
| 34 | STAT5B | na | 18496 | -0.008 | 0.1371 | No |
| 35 | MYC | na | 19651 | -0.019 | 0.1064 | No |
| 36 | CHUK | na | 20245 | -0.024 | 0.0927 | No |
| 37 | CCND1 | na | 20546 | -0.027 | 0.0879 | No |
| 38 | MAP2K2 | na | 20653 | -0.028 | 0.0888 | No |
| 39 | PIK3CB | na | 20685 | -0.028 | 0.0920 | No |
| 40 | PIK3CA | na | 21359 | -0.036 | 0.0776 | No |
| 41 | TCF7L2 | na | 22134 | -0.044 | 0.0615 | No |
| 42 | PIK3R2 | na | 22209 | -0.045 | 0.0658 | No |
| 43 | SOS2 | na | 22550 | -0.049 | 0.0629 | No |
| 44 | RPS6KB1 | na | 22694 | -0.051 | 0.0659 | No |
| 45 | RPS6KB2 | na | 23175 | -0.057 | 0.0602 | No |
| 46 | IKBKB | na | 23356 | -0.060 | 0.0634 | No |
| 47 | CEBPA | na | 24708 | -0.081 | 0.0359 | No |
| 48 | BRAF | na | 25048 | -0.086 | 0.0383 | No |
| 49 | KRAS | na | 25083 | -0.087 | 0.0496 | No |
| 50 | MTOR | na | 25211 | -0.089 | 0.0585 | No |
| 51 | PIK3CG | na | 26210 | -0.109 | 0.0451 | No |
| 52 | SPI1 | na | 26751 | -0.120 | 0.0465 | No |
| 53 | SOS1 | na | 26852 | -0.122 | 0.0609 | No |
| 54 | AKT2 | na | 27111 | -0.128 | 0.0715 | No |
| 55 | PIK3R1 | na | 27720 | -0.144 | 0.0743 | No |
| 56 | FLT3 | na | 31973 | -0.292 | -0.0070 | No |
| 57 | PIK3R5 | na | 34383 | -0.611 | 0.0100 | No |
Table: GSEA details [plain text format]

  

Fig 2: KEGG\_ACUTE\_MYELOID\_LEUKEMIA      
 Blue-Pink O' Gram in the Space of the Analyzed GeneSet

  

Fig 3: KEGG\_ACUTE\_MYELOID\_LEUKEMIA: Random ES distribution      
 Gene set null distribution of ES for **KEGG\_ACUTE\_MYELOID\_LEUKEMIA**
